# Supplementary material for: An integrated genome-wide multi-omics analysis of gene expression dynamics in the preimplantation mouse embryo
Source: Sci Rep. 2019 Sep 16;9:13356. doi: 10.1038/s41598-019-49817-3 (PMC6746714; doi:10.1038/s41598-019-49817-3)
Supplement: Supplementary file 1 — Supplementary Methods [file 41598_2019_49817_MOESM1_ESM.pdf]

# An integrated genome-wide multi-omics analysis of gene expression dynamics in the preimplantation mouse embryo

Steffen Israel[1,*], Mathias Ernst[2,3,*], Olympia E. Psathaki[4], Hannes C. A. Drexler[1], Ellen Casser[1], Yutaka Suzuki[5], Wojciech Makalowski[6], Michele Boiani[1,†], Georg Fuellen[2,†], Leila Taher[2,3,†]

[1] Max-Planck-Institute for Molecular Biomedicine, Roentgenstr. 20, 48149 Muenster, Germany

[2] Institute for Biostatistics and Informatics in Medicine and Ageing Research, Rostock University Medical Center, Ernst-Heydemann Str. 8, 18057 Rostock, Germany

[3] Bioinformatics, Department of Biology, Friedrich-Alexander-Universität Erlangen-Nürnberg, Staudtstr. 5, 91058 Erlangen, Germany

[4] University of Osnabrück, Center for Cellular Nanoanalytics Osnabrück (CellNanOs), Integrated Bioimaging Facility Osnabrück (iBiOs), Barbarastr. 11, 49076 Osnabrück, Germany

[5] Department of Medical Genome Sciences, Graduate School of Frontier Sciences, University of Tokyo, Kashiwa, Chiba, 277-8562, Japan

[6] Institute of Bioinformatics, Faculty of Medicine, University of Muenster, Niels Stensen Str. 14, 48149, Muenster, Germany

* These authors contributed equally

† Corresponding authors: LT: leila.taher@fau.de; GF: fuellen@uni-rostock.de; MB: mboiani@mpi-muenster.mpg.de.

**Running Title:** Multi-omics of the preimplantation mouse embryo

**Keywords:** Preimplantation development, Proteome, Transcriptome, Model Organism

Supplementary Methods .......................................................................................................... 3
References .......................................................................................................... 12

## Supplementary Methods

### MS/MS spectra search against mouse protein database

MS/MS spectra were searched against the mouse UniprotKB database (version from Dec. 2015, [1]) concatenated with reversed sequence versions of all entries and supplemented with common contaminants. Parameters defined for the search were trypsin as the digesting enzyme, allowing two missed cleavages; a minimum length of seven amino acids; carbamidomethylation at cysteine residues as fixed modification, oxidation at methionine and protein N-terminal acetylation as variable modifications. The maximum allowed mass deviation was 20 ppm for the MS and 0.5 Da for the MS/MS scans. Protein groups were identified with a false discovery rate set to 1% for all peptide and protein identifications separately, when there were at least two matching peptides, at least one of which was unique to the protein group.

### Proteome and transcriptome clustering

First, for each protein detected at least at two developmental stages in at least two replicates we computed a linear model:

$$\log_2 \frac{L}{H} = \mu + T_i + \epsilon$$

where $\mu$ is the global mean for the gene, $T_i$ is a categorical explanatory variable representing the developmental stage, and $\epsilon$ denotes the error. Next, we used the ANOVA P-value corresponding to $T_i$ as filtering criterion, and retained only those proteins for which P-value $\leq$ 0.05. Additionally, we required the proteins to have a difference of at least 2 between the largest and the smallest $\log_2 \frac{L}{H}$ ratio across all developmental stages. This resulted in 764 proteins.

We clustered the ($\log_2$) fold-change of the protein L/H ratios relative to the oocyte. ($\log_2$) fold-changes were centered and scaled before performing clustering with the R package Mfuzz [2,3]. For each resulting cluster we then constructed a graph with nodes representing the members of the cluster and edges representing Pearson correlation coefficients between the expression profiles of the proteins associated with the nodes such that $r^2 \geq 0.5$. Finally, only those proteins within complete subgraphs (cliques) were selected as final members of the cluster.

We clustered the cognate transcripts of the aforementioned 764 proteins into seven clusters following an analogous procedure. The 2-cell stage represents the average of the ($\log_2$) fold-changes between the early and late 2-cell stages.

Overlap between protein and transcript clusters

The significance of the overlap between the members of all possible pairs of protein and transcript clusters (based on their official gene symbols) was computed using a Fisher's exact test with the 764 proteins/transcripts initially subjected to clustering serving as background.

## Functional enrichment analysis

Gene Ontology (GO) enrichment analysis was performed using the Database for Annotation and Integrated Discovery (DAVID, Version 6.8, [4,5]). Proteins and transcripts were submitted to DAVID using Ensembl IDs specifying *Mus musculus* as the species. Significantly overrepresented biological process (BP), molecular function (MF) and cellular component (CC) GO terms were retrieved by using the options GOTERM_BP_ALL, GOTERM_BP_DIRECT, GOTERM_BP_FAT, GOTERM_CC_ALL, GOTERM_CC_DIRECT, GOTERM_CC_FAT, GOTERM_MF_ALL, GOTERM_MF_DIRECT, GOTERM_MF_FAT, KEGG_PATHWAY, UP_KEYWORDS, and UP_TISSUE. The default parameters and corresponding false discovery rate (FDR) by the Benjamini and Hochberg approach [6] were used to determine significant enrichment.

In this context, we refer to the theoretical proteome/transcriptome as the entire potential protein complement encoded by the genome of the mouse, and distinguish it from the detected proteome/transcriptome, comprising only the proteins/transcripts detected in at least one replicate. For each pair of protein and transcript clusters, GO term associations for shared proteins/transcripts were tested for significance against a background set consisting of the members of the corresponding protein cluster.

## Concordance between the proteome and the transcriptome

For every pair of developmental stages $S_i$ and $S_j$ we separated the proteins into two groups according to the change in expression (up- or down-regulation) of their cognate transcripts at $S_i$ relative to the oocyte. At each developmental stage, only proteins detected in at least two of the replicates and of the oocyte and their corresponding transcripts were considered. A gene was considered up-regulated if it was significantly differentially expressed (see Methods) and exhibited a fold-change $\geq 2$; on the contrary, a gene was considered down-regulated if it was significantly differentially expressed (see Methods) and exhibited a fold-change $\leq 0.5$. For each of the two resulting groups of proteins, we estimated the cumulative distribution function (CDF) of their ($\log_2$) fold-changes at $S_j$ relative to the oocyte using the ecdf() function in R. Thus, for each pair of developmental time points, we estimated two such CDFs: one for the proteins whose transcripts are up-regulated and one for the proteins whose transcripts are down-regulated. Finally, we integrated the two CDFs between the minimum and maximum ($\log_2$) fold-changes as determined above, yielding two areas under the curve (AUCs). To quantify the shift between the CDFs we subtracted the AUC for the proteins whose transcripts are up-regulated from the AUC for the proteins whose transcripts are down-regulated. For robustness, only sets containing at least 25 proteins/transcripts were used for calculating the AUC. This approach is common in other contexts, such as drug discovery, and has been used to describe the relationship between the transcriptome and the proteome, for example, in the ageing rat [7]. For the transcriptome, the 2-cell stage represents the average of the ($\log_2$) fold-changes between the early and late 2-cell stages; a transcript is considered differentially expressed at the 2-cell stage if it is differentially expressed at the early or late 2-cell stage relative to the oocyte.

## Number of clusters

Note that the number of clusters $k$ is a parameter of the algorithm. We therefore performed fuzzy clustering for $k \in \{4, 5, 6, 7, 8\}$ and examined the resulting membership matrices, which specify the probability with which each protein/transcript is assigned to each cluster. Specifically, we computed a PCA on this matrix using the clusters as features. We then determined the members of each cluster (based on the hard-clustering) and computed an ellipse

(using the function ellipse() in the R package ellipse with default parameters) defined by the covariance matrix of the cluster PC scores and centered on the mean of the PC scores. Finally, we visually inspected the PCA plot derived from the membership matrix with the ellipses for each cluster overlaid on top. We chose the largest value of *k* for which the ellipses are clearly separated from each other (see Figures below).

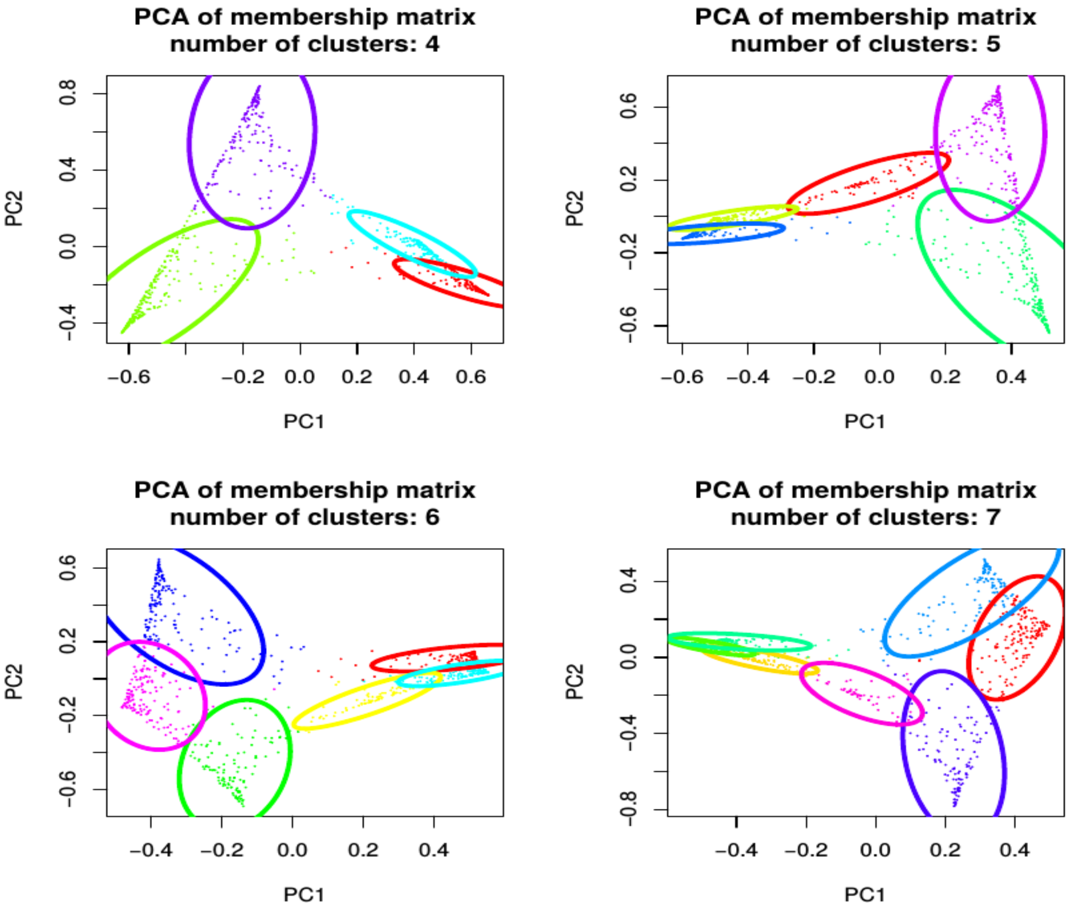


**Number of protein clusters.** We performed fuzzy clustering on the protein profiles. Like k-means, the fuzzy clustering algorithm requires the number of clusters *k* to be set in advance. We performed clustering for *k*=4, 5, 6 and 7 and principal component analysis (PCA) on each of the resulting membership matrices. The figures show the first two PCs. The ellipses are based on the mean and the covariance matrix of each cluster. A clear separation between the ellipses is indicative of distinct clusters. We chose the highest *k* for which the ellipses were clearly separated, in this case, 6. Note than when enforcing seven clusters (bottom right panel), the

seventh one is wedged in between two preexisting ones (upper left portion of the subfigure, note that orientation and coloring are arbitrary, thus not comparable between subfigures).

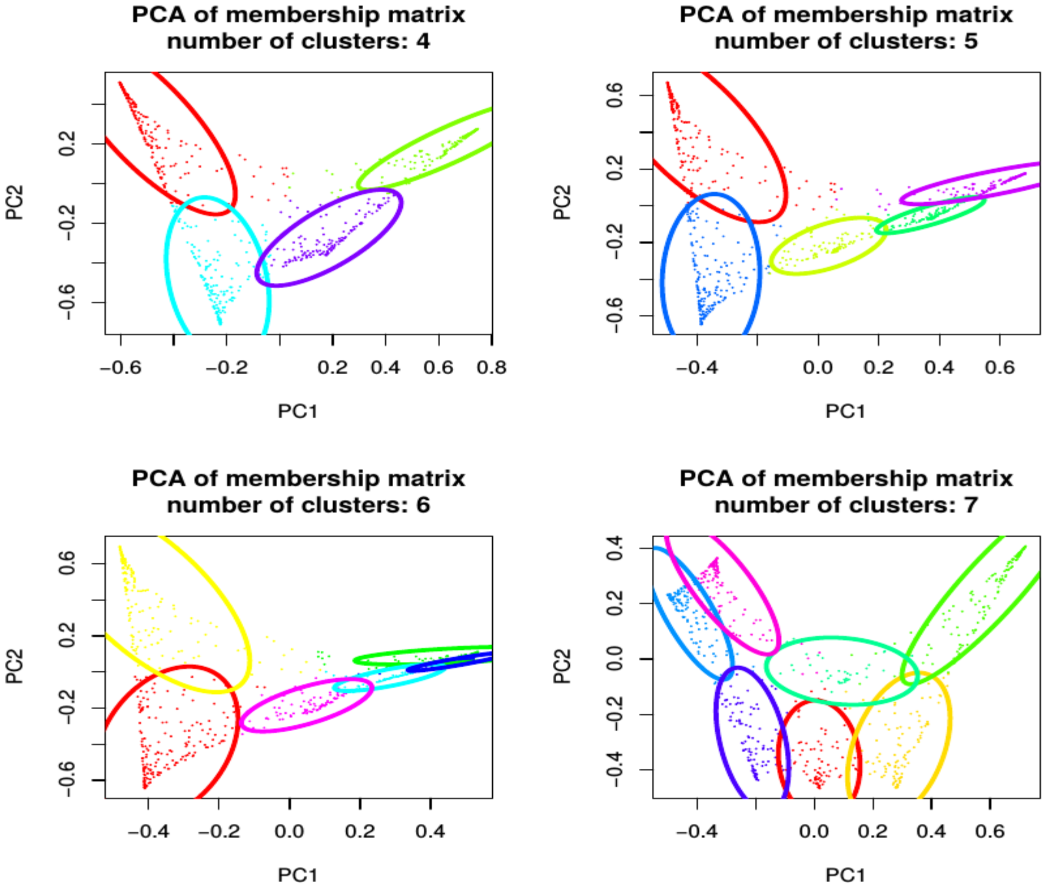


**Number of transcript clusters.** See figure above for details. Best separation is observed for 7 clusters.

## Cluster profiles

The profile of each cluster was defined by computing the median expression value across its members for each developmental stage.

## Markers for early and late preimplantation developmental stages

This analysis was performed using the proteins detected in all replicates of all developmental stages (1,709 proteins). Given $N_1$ samples from sample group $S_i$ and $N_j$ samples

from sample group $S_j$, the classification problem is to predict the sample group (class) of any other sample based only on its protein $\log_2$ L/H ratios. For the evaluation of the classification models we used leave-one-out cross-validation, meaning that at each iteration of the cross-validation procedure, each of the $N_i + N_j$ samples was used for testing exactly once, while the remaining $N_i + N_j - 1$ samples were used for training. Thus, for a pair of sample groups $S_i$ and $S_j$, we trained and tested $N_i + N_j$ models. For producing the results reported here we grouped the developmental stages into two sample groups: (i) early (oocyte, 1- and 2-cell-embryo stages, $N_1 = 3 + 3 + 3 = 9$) and late preimplantation development (4- and 8-cell, morula and blastocyst embryo stages, $N_1 = 3 + 3 + 3 + 2 = 11$), and trained a total of 20 classifiers. The reported classification rate is the fraction of correctly classified samples among the $20$ tests.

At each iteration of the cross-validation procedure, we selected features with a mean $\log_2$ L/H ratio $\geq 1$ in at least one of the two classes in the training set and discarded the rest from further analysis. Next, we performed Principal Component Analysis (PCA). To regularize the classification problem and avoid over-fitting of the classifier, only the first two principal components (PC1 and PC2) were used as features to train a Linear Discriminant Analysis (LDA) model. The aim of LDA is to find a hyperplane that separates between two classes based on their features.

To classify the test sample we first projected its $\log_2$ L/H ratios onto PC1 and PC2 by multiplying the zero-centered $\log_2$ L/H ratios with the PCA rotation matrix and keeping only the first two components, and assigned a class based on the aforementioned hyperplane. If the LDA did not achieve a perfect class separation for the training sample, the classification of the test sample was considered “incorrect” and the classification rate computed accordingly.

Finally, we identified the features that were among the most discriminant features at all iterations of the cross-validation procedure. A protein ranking was thus extracted based on the LDA. Specifically, we projected PC1 and PC2 onto the LDA space. This projection is a linear combination of PC1 and PC2, which are in turn linear combinations of the original protein $\log_2$ L/H ratios. Hence, the most discriminant proteins are those with the highest and lowest coefficients in the linear combination. We computed a rank for each classifier and aggregated them using the RankAggregg() function in the RankAggregg R package [8]. From this aggregated ranking, we selected the top 20 proteins as the most discriminant features between the two classes.

## Comparison to other published proteomic datasets

We compared our proteomic data to that of Gao et al. [9]. We obtained the data from the available spreadsheet (Embryo1" and "Embryo2" in Table S1). Specifically, we worked with the columns P to U, which correspond to normalized ratios for the six developmental stages that were analyzed in their study (from zygote to blastocyst). We used the org.Mm.eg.db R/BioConductor package (version 3.4.1, [10]) to identify (and translate, if necessary) the official symbol of the proteins provided in column V. In approximately 10% of the cases, the reported symbols were aliases. Entries in the original dataset associated with multiple official symbols were discarded. Replicates were collapsed by computing the mean of the two replicates or the single available value in cases in which the protein had only been detected in one replicate. For the sake of compatibility, we excluded our oocyte data from the comparison.

## Protein complexes

The definition of the protein complexes was obtained from the resource compiled by Ori et al [11]. Specifically, we downloaded Additional file 2, which comprises the descriptions of 279 protein complexes, including the human Ensembl gene identifier of their members. Human Ensembl gene identifiers were mapped to mouse orthologs using the Ensembl orthology data [12,13] and the BioMart interface of Ensembl [14]. Out of a total of 279 protein complexes, 233 had 5 or more mouse members and were considered for the analysis.

## Validation of candidate protein markers

The twenty candidate protein markers were validated using two proteomics datasets (see Supplemental Table S5). The data from Gao et al. [9] was processed as described before (see "Comparison to other published proteomic datasets"). Furthermore, we generated an additional SILAC dataset. Metaphase II (MII) and fertilized oocytes were collected from the oviductal ampullae of 6-8 week-old B6C3F1 mice without prior ovarian stimulation. Females were mated to vasectomized or stud CD1 males to collect MII or fertilized oocytes, respectively, and

euthanized by cervical dislocation. MII oocytes were lysed immediately in SDS buffer. Fertilized oocytes were cultured in KSOM(aa) medium at 37 °C in a humidified atmosphere of 6 % CO2 in air. Developmental stages were collected from the medium based on morphology at the appropriate time points (defined as time after mating, which in case of ovarian stimulation is the same as time after administration of hCG): 1-cell stage, 16 hours; 2-cell stage, 43 hours; 4-cell stage, 53 hours; 8-cell stage, 62 hours; morula, 72 hours; blastocyst, 92 hours). Before lysing in SDS buffer, the oocytes' zona pellucida was removed using acidic Tyrode's solution so as to increase the sensitivity of proteome analysis for the other oocytic proteins. The LC-MSMS pipeline was based on the stable isotope labeling of a F9 embryonal carcinoma (EC) cells as spike-in reference for the oocyte samples. In brief, proteins from zona-free oocytes or embryos were mixed in a 1:1 ratio (protein amount) with the heavy F9 carcinoma spike-in cell lysate (Lys8 and Arg10), acetone-precipitated, reduced (DTT) and alkylated JAA), and then digested with Endoproteinase Lys-C (3hr) and Trypsin (overnight). Following desalting on Empore 3M C18 discs, peptide mixtures were offline fractionated by RP-HPLC at pH 10.2 (Buffer A: 10mM ammonium formate pH 10.2; Buffer B: 10mM ammonium formate, 90% acetonitril, pH 10.2; linear gradient from 0-35% B in 70min; 35 - 70% B in 15min; 70% B for 10min; Waters XBridge BEH C18 2.1 x 150mm). Twenty pools were generated from each sample by concatenated fractionation, lyophilized and subsequently analyzed individually by LC-MS/MS either on a LTQ Orbitrap Velos (experiment 0746) or on a Q Exactive (experiments 0860, 911) mass spectrometer (Thermo Scientific, Waltham, MA 02454, USA) both equipped with an Easy nano-LC system and a nano-electrospray source (both from Proxeon, Odense, Denmark) holding 15 cm fused silica capillary emitter columns (New Objective, ID 75µm) filled with a C18 reversed phase matrix (ReproSil-Pur C18-AQ, 3µm; Dr. Maisch, Ammerbuch). Both mass spectrometers were operated in data-dependent mode (positive ion mode, source voltage 2.1kV) automatically switching between a survey scan (Orbitrap Velos: mass range m/z = 350-1650, target value = 1 x 106; resolution R = 60 K; lock mass set to background ion 445.120025; Q Exactive: mass range 300 – 1750; target value = 3 x 106; resolution 70K) and MS/MS acquisition of the 15 (Velos) and 10 (Q Exactive) most intense peaks by collisional induced dissociation (CID) in the ion trap in case of the Velos (isolation width m/z = 2.0; normalized collision energy 35%; dynamic exclusion enabled with repeat count 1, repeat duration 30.0, exclusion list size 500 and exclusion duration set to 90 s) or by higher energy induced collisional

dissociation (HCD) in case of the Q Exactive (isolation width m/z = 1.6; normalized collision energy 25%; dynamic exclusion enabled and set to 25.0 s); double charge and higher charges were allowed. Gradient conditions for the reversed-phase online separation of peptide mixtures were 2-28% buffer B (80% acetonitril, 0.1% formic acid; 120min), 28-98% B (20min), 98% B (6min) for the Orbitrap Velos; and 2-30% B (120min), 30-50% B (30min), 50-95% B (5min), 95% B (5min) for the Q Exactive. Afterwards, columns were re-equilibrated in Buffer A (0.1% formic acid). The MS proteomics data have been deposited to the ProteomeXchange Consortium (http://proteomecentral.proteomexchange.org) via the PRIDE partner repository [15].

## References


1 The UniProt, C. UniProt: the universal protein knowledgebase. *Nucleic Acids Res* **45**, D158-D169, doi:10.1093/nar/gkw1099 (2017).

2 Futschik, M. E. & Carlisle, B. Noise-robust soft clustering of gene expression time-course data. *J Bioinform Comput Biol* **3**, 965-988 (2005).

3 Kumar, L. & M, E. F. Mfuzz: a software package for soft clustering of microarray data. *Bioinformation* **2**, 5-7 (2007).

4 Huang da, W., Sherman, B. T. & Lempicki, R. A. Systematic and integrative analysis of large gene lists using DAVID bioinformatics resources. *Nat Protoc* **4**, 44-57, doi:10.1038/nprot.2008.211 (2009).

5 Huang da, W., Sherman, B. T. & Lempicki, R. A. Bioinformatics enrichment tools: paths toward the comprehensive functional analysis of large gene lists. *Nucleic Acids Res* **37**, 1-13, doi:10.1093/nar/gkn923 (2009).

6 Benjamini, Y. & Hochberg, Y. Controlling the false discovery rate: a practical and powerful approach to multiple testing. *Journal of the Royal Statistical Society. Series B* **57**, 289-300 (1995).

7 Ori, A. *et al.* Integrated Transcriptome and Proteome Analyses Reveal Organ-Specific Proteome Deterioration in Old Rats. *Cell Syst* **1**, 224-237, doi:10.1016/j.cels.2015.08.012 (2015).

8 Pihur, V., Datta, S. & Datta, S. RankAggreg, an R package for weighted rank aggregation. *BMC Bioinformatics* **10**, 62, doi:10.1186/1471-2105-10-62 (2009).

9 Gao, Y. *et al.* Protein Expression Landscape of Mouse Embryos during Pre-implantation Development. *Cell Rep* **21**, 3957-3969, doi:10.1016/j.celrep.2017.11.111 (2017).

10 org.Mm.eg.db: Genome wide annotation for Mouse. R package version 3.6.0. (2018).

11 Ori, A. *et al.* Spatiotemporal variation of mammalian protein complex stoichiometries. *Genome Biol* **17**, 47, doi:10.1186/s13059-016-0912-5 (2016).

12 Vilella, A. J. *et al.* EnsemblCompara GeneTrees: Complete, duplication-aware phylogenetic trees in vertebrates. *Genome research* **19**, 327-335, doi:10.1101/gr.073585.107 (2009).

13 Flicek, P. *et al.* Ensembl 2014. *Nucleic Acids Res* **42**, D749-755, doi:10.1093/nar/gkt1196 (2014).

14 Kinsella, R. J. *et al.* Ensembl BioMarts: a hub for data retrieval across taxonomic space. *Database (Oxford)* **2011**, bar030, doi:10.1093/database/bar030 (2011).

15 Vizcaino, J. A. *et al.* The PRoteomics IDEntifications (PRIDE) database and associated tools: status in 2013. *Nucleic Acids Res* **41**, D1063-1069, doi:10.1093/nar/gks1262 (2013).
