# Supplementary material for: An integrated genome-wide multi-omics analysis of gene expression dynamics in the preimplantation mouse embryo
Source: Sci Rep. 2019 Sep 16;9:13356. doi: 10.1038/s41598-019-49817-3 (PMC6746714; doi:10.1038/s41598-019-49817-3)
Supplement: Supplementary file 2 — Supplementary Figures and Table Legends [file 41598_2019_49817_MOESM2_ESM.pdf]

An integrated genome-wide multi-omics analysis of gene expression dynamics in the preimplantation mouse embryo

Steffen Israel[1,*], Mathias Ernst[2,3,*], Olympia E. Psathaki[4], Hannes C. A. Drexler[1], Ellen Casser[1], Yutaka Suzuki[5], Wojciech Makalowski[6], Michele Boiani[1,†], Georg Fuellen[2,†], Leila Taher[2,3,†]

[1] Max-Planck-Institute for Molecular Biomedicine, Roentgenstr. 20, 48149 Muenster, Germany

[2] Institute for Biostatistics and Informatics in Medicine and Ageing Research, Rostock University Medical Center, Ernst-Heydemann Str. 8, 18057 Rostock, Germany

[3] Bioinformatics, Department of Biology, Friedrich-Alexander-Universität Erlangen-Nürnberg, Staudtstr. 5, 91058 Erlangen, Germany

[4] University of Osnabrück, Center for Cellular Nanoanalytics Osnabrück (CellNanOs), Integrated Bioimaging Facility Osnabrück (iBiOs), Barbarastr. 11, 49076 Osnabrück, Germany

[5] Department of Medical Genome Sciences, Graduate School of Frontier Sciences, University of Tokyo, Kashiwa, Chiba, 277-8562, Japan

[6] Institute of Bioinformatics, Faculty of Medicine, University of Muenster, Niels Stensen Str. 14, 48149, Muenster, Germany

* These authors contributed equally

† Corresponding authors: LT: leila.taher@fau.de; GF: fuellen@uni-rostock.de; MB: mboiani@mpi-muenster.mpg.de.

Running Title: Multi-omics of the preimplantation mouse embryo

Keywords: Preimplantation development, Proteome, Transcriptome, Model Organism

Supplementary Figures 3
Supplementary Table Legends.. 26
References 27

Supplementary Figures

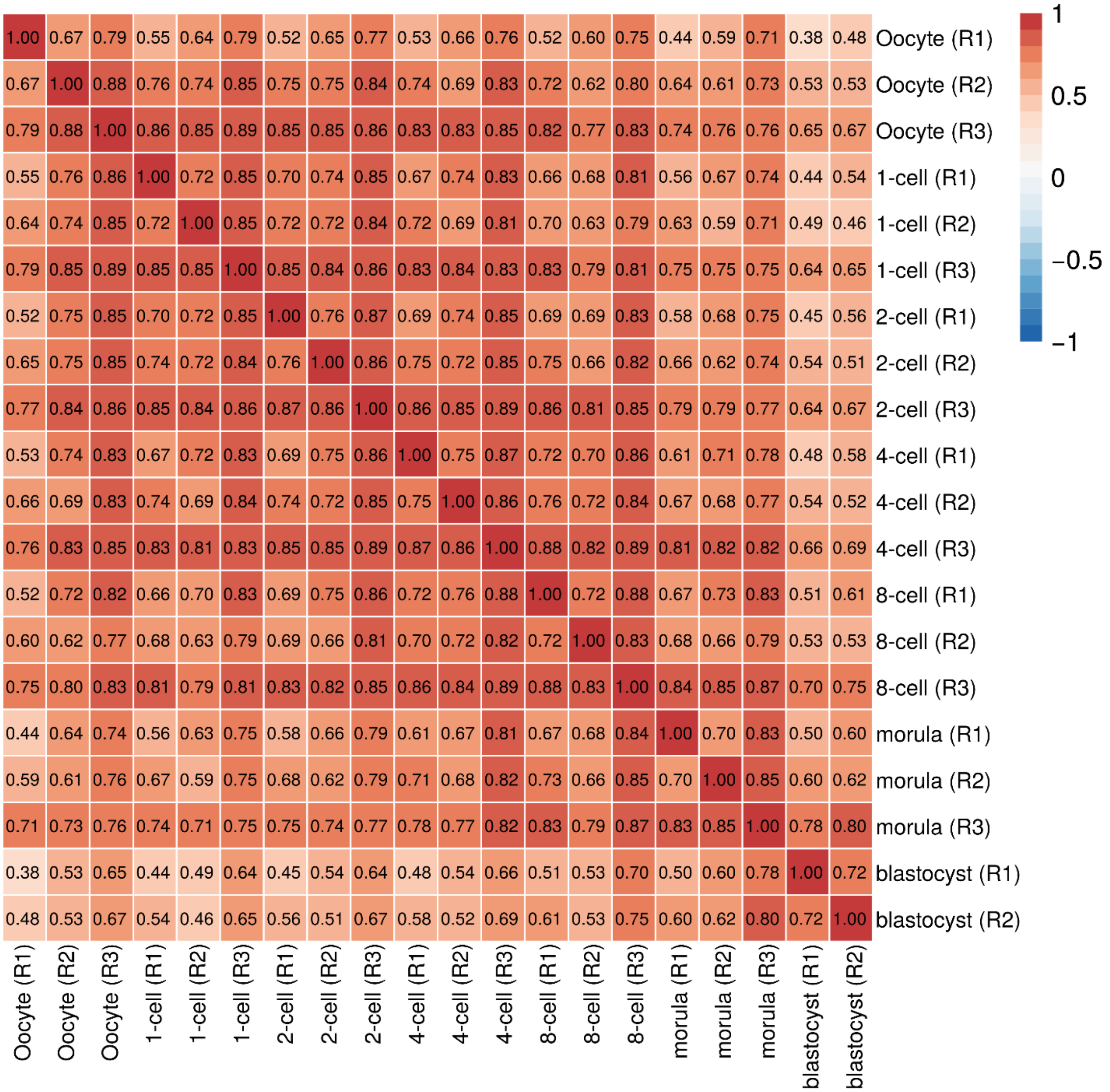

Supplemental Fig. S1. Proteome replicates are strongly correlated. Spearman's rank correlations of pairwise comparisons between the protein (log_2) L/H ratios of the 20 samples in the study.

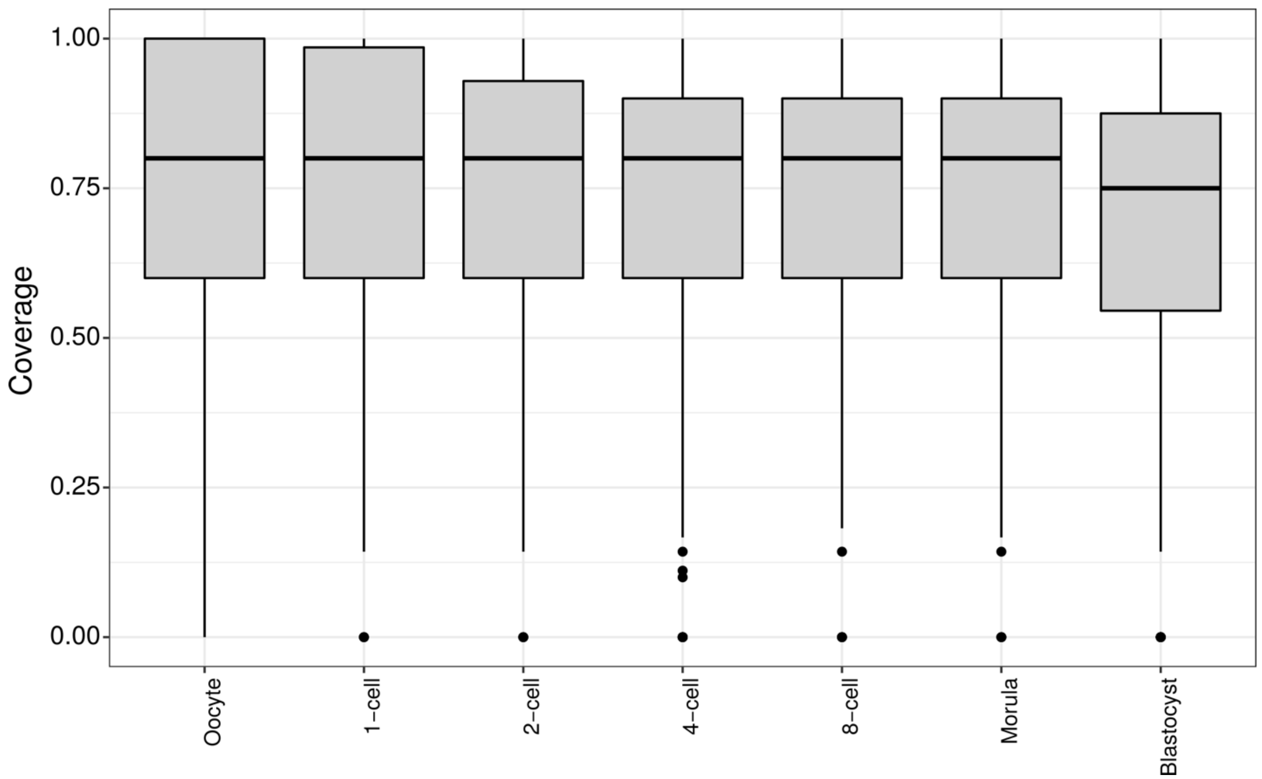

Supplemental Fig. S2. Coverage distribution for 233 protein complexes across different development stages. Complexes comprise 5 to 153 protein members (with a median of 7). The coverage was computed as the fraction of the members of a complex detected in at least one of the replicates of the developmental stage under consideration. The distributions of the fractions are shown as boxplots.

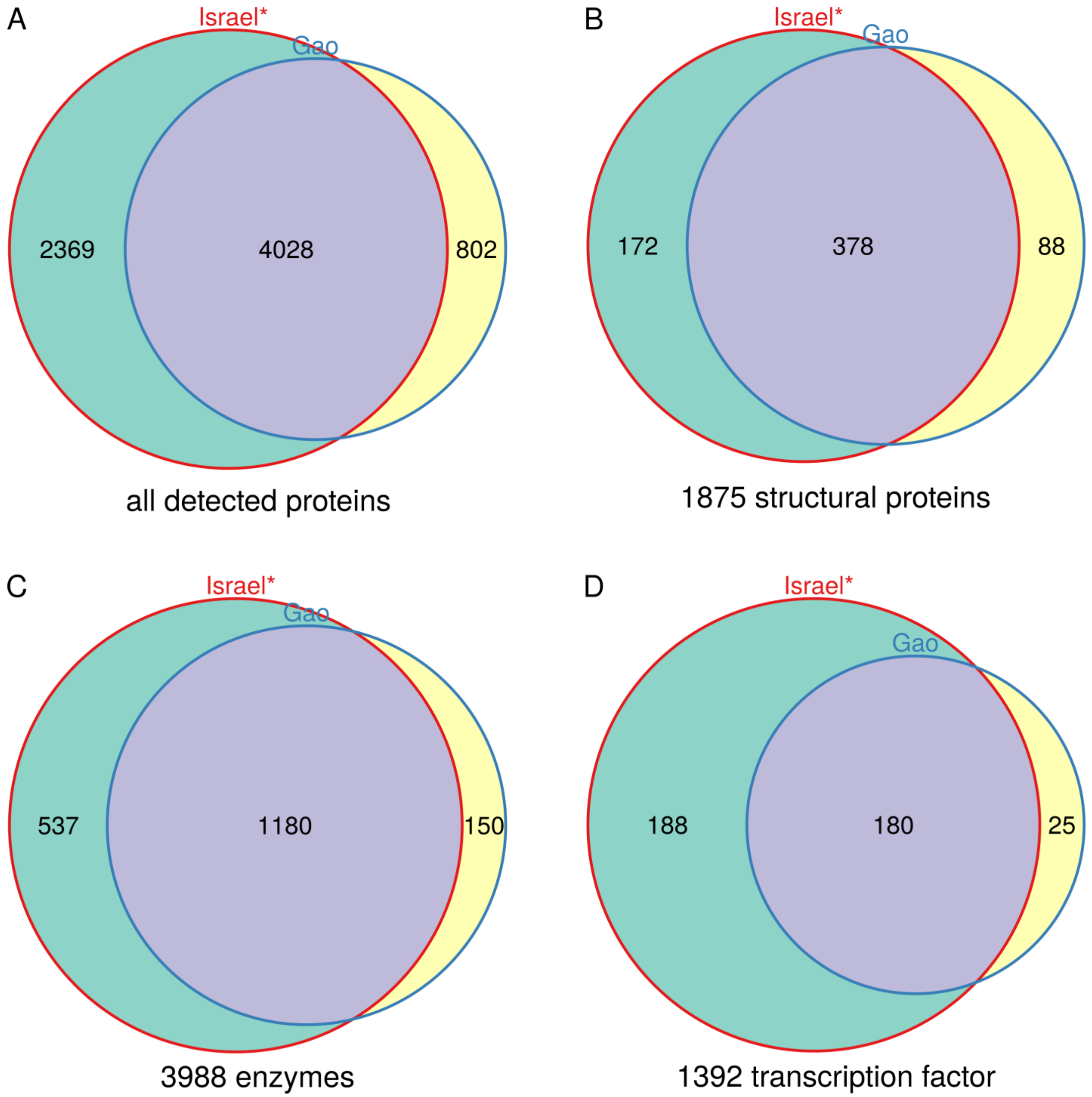

Supplemental Fig. S3. Proteins detected in at least one replicate of at least one developmental stage (zygote to blastocyst only) in our dataset and in Gao et al's dataset [1]. A) all proteins. B-C) Structural proteins, enzymes and transcription factors, defined based on the annotation of the PANTHER Classification System (http://www.pantherdb.org/, [2,3]). Structural proteins (B) were defined as proteins comprised in the PANTHER classes "cytoskeletal protein" (PC00085), "cell junction protein" (PC00070), "structural protein" (PC00211), "membrane traffic protein" (PC00150), "extracellular matrix protein" (PC00102), "cell adhesion molecule" (PC00069) and "viral coat protein" (PC00236) and all PANTHER classes that are children and descendants of these classes in the hierarchy of the PANTHER database. 1,875 proteins among the complete mouse proteome can be classified as "structural proteins" in this manner. Enzymes (C) were defined as proteins comprised in PANTHER classes with names including the "-ase"

suffix and its children and descendants. 3,988 proteins among the complete mouse proteome can be classified as “enzymes” in this manner. Transcription factors (D) were defined as proteins comprised in the PANTHER class “transcription factor” (PC00218) and all its children and descendants. 1,392 proteins among the complete mouse proteome can be classified as “transcription factors” in this manner. The Venn diagram (generated using version 3.0 of the Vennerable R package) indicates the number of proteins detected in our dataset (Israel*) and in Gao et al’s dataset (Gao)[1]. Note that we excluded our oocyte data for comparability.

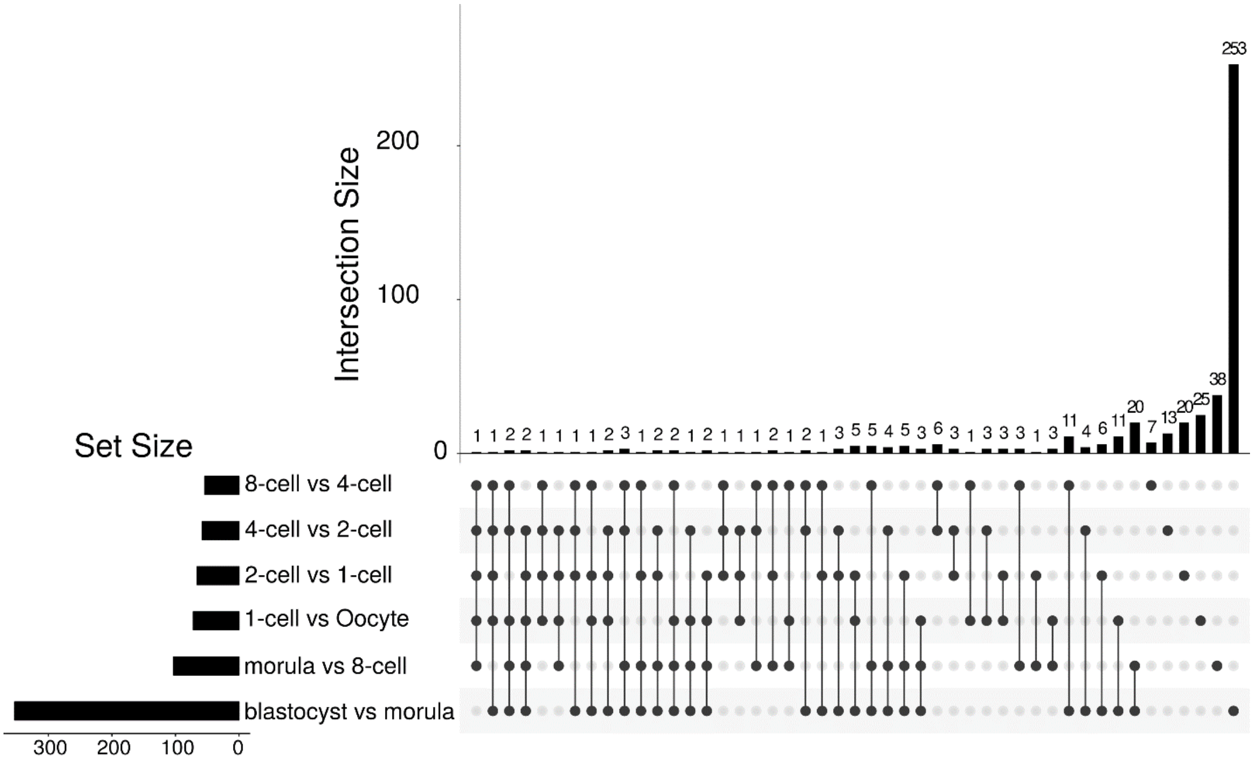

Supplemental Fig. S4. UpSet plots [4] showing the overlap between the sets of differentially expressed proteins between pairs of consecutive developmental stages (fold-change ≥2 or ≤ 0.5, P-value ≤ 0.05 from ANOVA). The bar represents the number of proteins shared by the pairs of stages indicated by the black dots and not by the stages indicated by the gray dots. A total of 488 proteins was differentially expressed between pairs of consecutive developmental stages; 356 were exclusively differentially expressed in one transition.

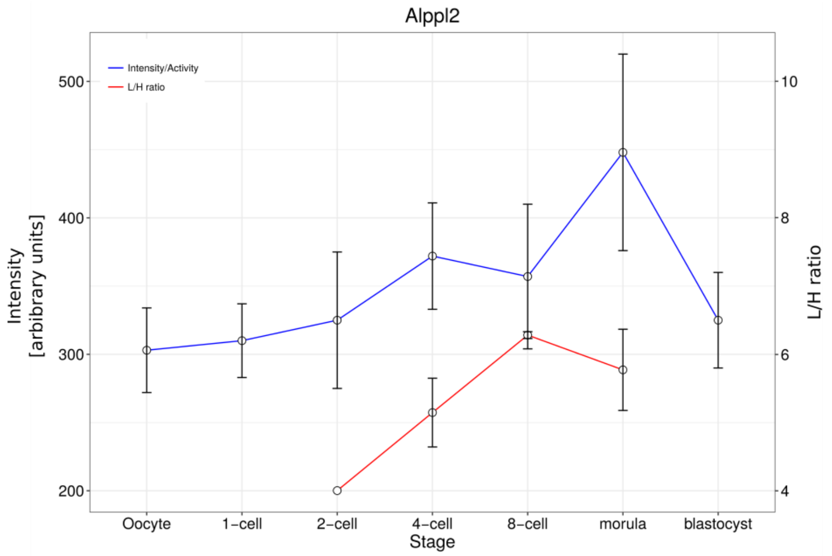
Alppl2
Intensity/Activity
L/H ratio
Intensity
[arbibrary units]
L/H ratio
500
400
300
200
10
8
6
4
Oocyte
1−cell
2−cell
4−cell
8−cell
morula
blastocyst
Stage

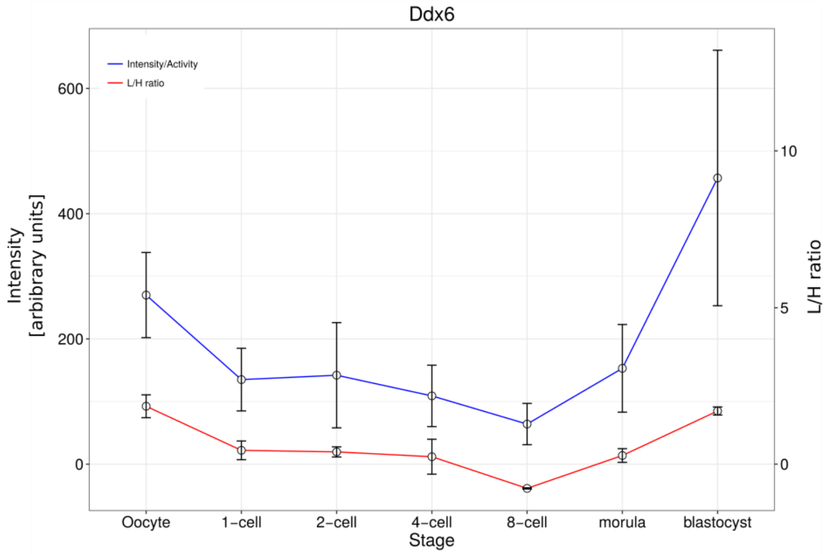
Ddx6
Intensity/Activity
L/H ratio
Intensity
[arbibrary units]
L/H ratio
600
400
200
0
10
5
0
Oocyte
1−cell
2−cell
4−cell
8−cell
morula
blastocyst
Stage

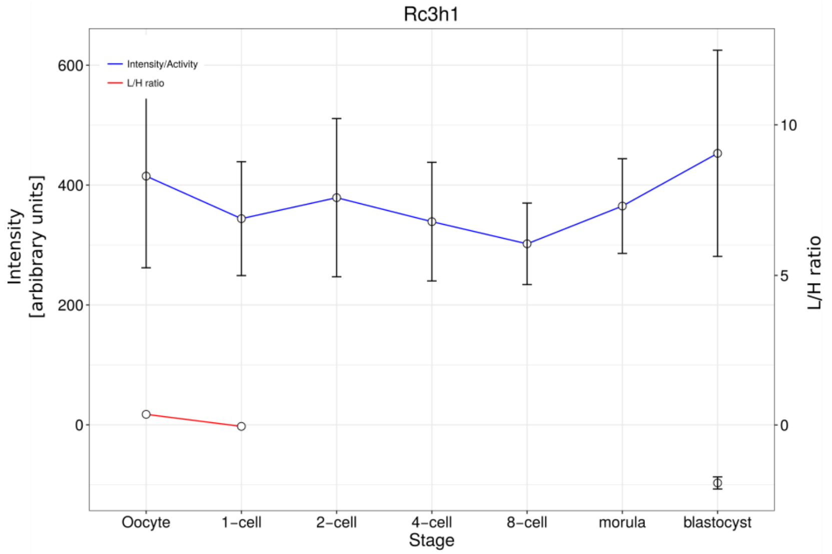
Rc3h1
Intensity/Activity
L/H ratio
Intensity
[arbibrary units]
L/H ratio
600
400
200
0
10
5
0
Oocyte
1−cell
2−cell
4−cell
8−cell
morula
blastocyst
Stage

Supplemental Fig. S5. Immunofluorescence validation of proteins levels. Mean and standard deviations of $\log_2$ L/H ratios L/H ratios and intensities of immunofluorescence images based on all available measurements. Intensities of immunofluorescence images are in arbitrary units.

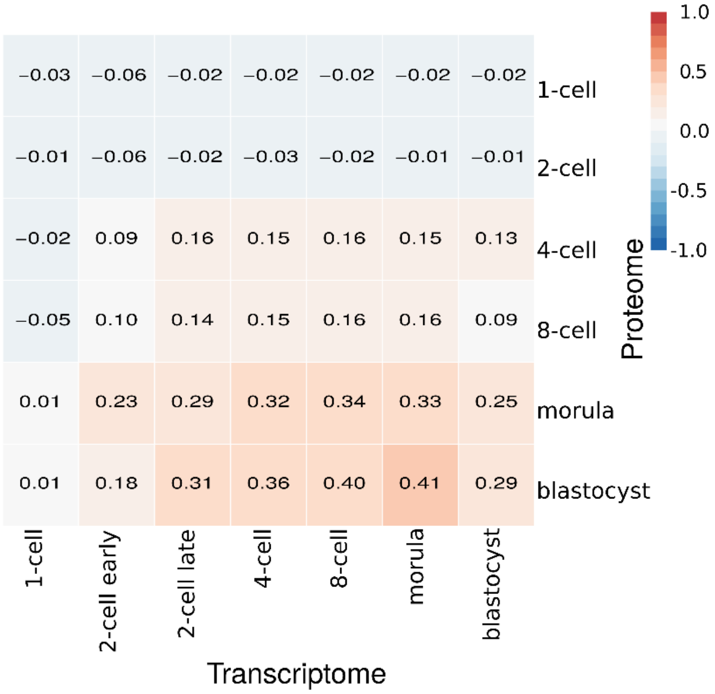

Supplemental Fig. S6. Spearman's rank correlations between the fold-changes in $\log_2$ L/H ratios and expression values observed for the proteins and for their cognate transcripts, respectively, relative to the oocyte. Sample group averages were considered for the calculation of pairwise correlations between the seven developmental stages in the study. Reported values are based on proteins detected in at least two replicates of each of the two developmental stages involved in each correlation calculation and their cognate transcript.

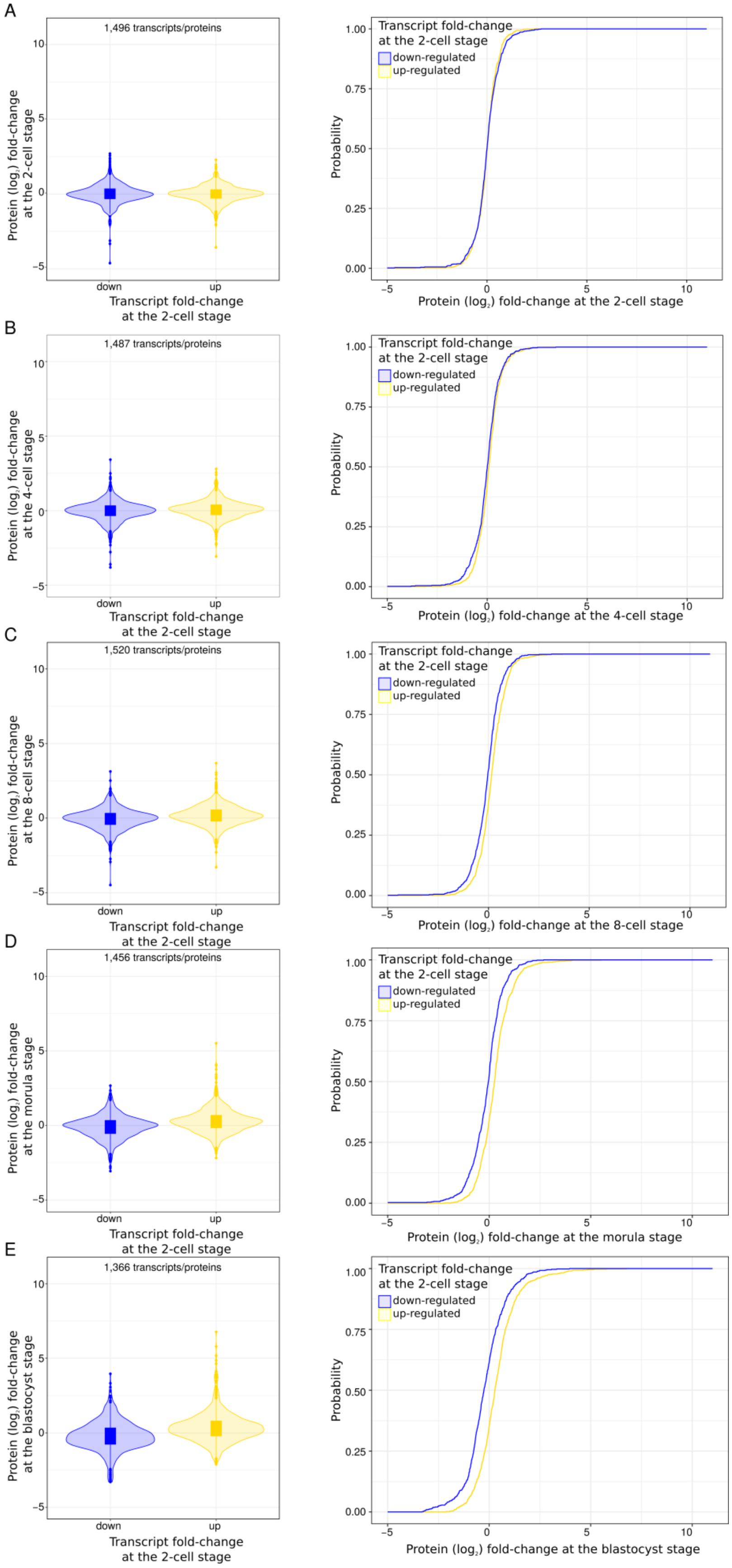
A
1,496 transcripts/proteins
Protein (log2) fold-change at the 2-cell stage
down
up
Transcript fold-change at the 2-cell stage
Transcript fold-change at the 2-cell stage
down-regulated
up-regulated
Probability
Protein (log2) fold-change at the 2-cell stage
B
1,487 transcripts/proteins
Protein (log2) fold-change at the 4-cell stage
down
up
Transcript fold-change at the 2-cell stage
Transcript fold-change at the 2-cell stage
down-regulated
up-regulated
Probability
Protein (log2) fold-change at the 4-cell stage
C
1,520 transcripts/proteins
Protein (log2) fold-change at the 8-cell stage
down
up
Transcript fold-change at the 2-cell stage
Transcript fold-change at the 2-cell stage
down-regulated
up-regulated
Probability
Protein (log2) fold-change at the 8-cell stage
D
1,456 transcripts/proteins
Protein (log2) fold-change at the morula stage
down
up
Transcript fold-change at the 2-cell stage
Transcript fold-change at the 2-cell stage
down-regulated
up-regulated
Probability
Protein (log2) fold-change at the morula stage
E
1,366 transcripts/proteins
Protein (log2) fold-change at the blastocyst stage
down
up
Transcript fold-change at the 2-cell stage
Transcript fold-change at the 2-cell stage
down-regulated
up-regulated
Probability
Protein (log2) fold-change at the blastocyst stage

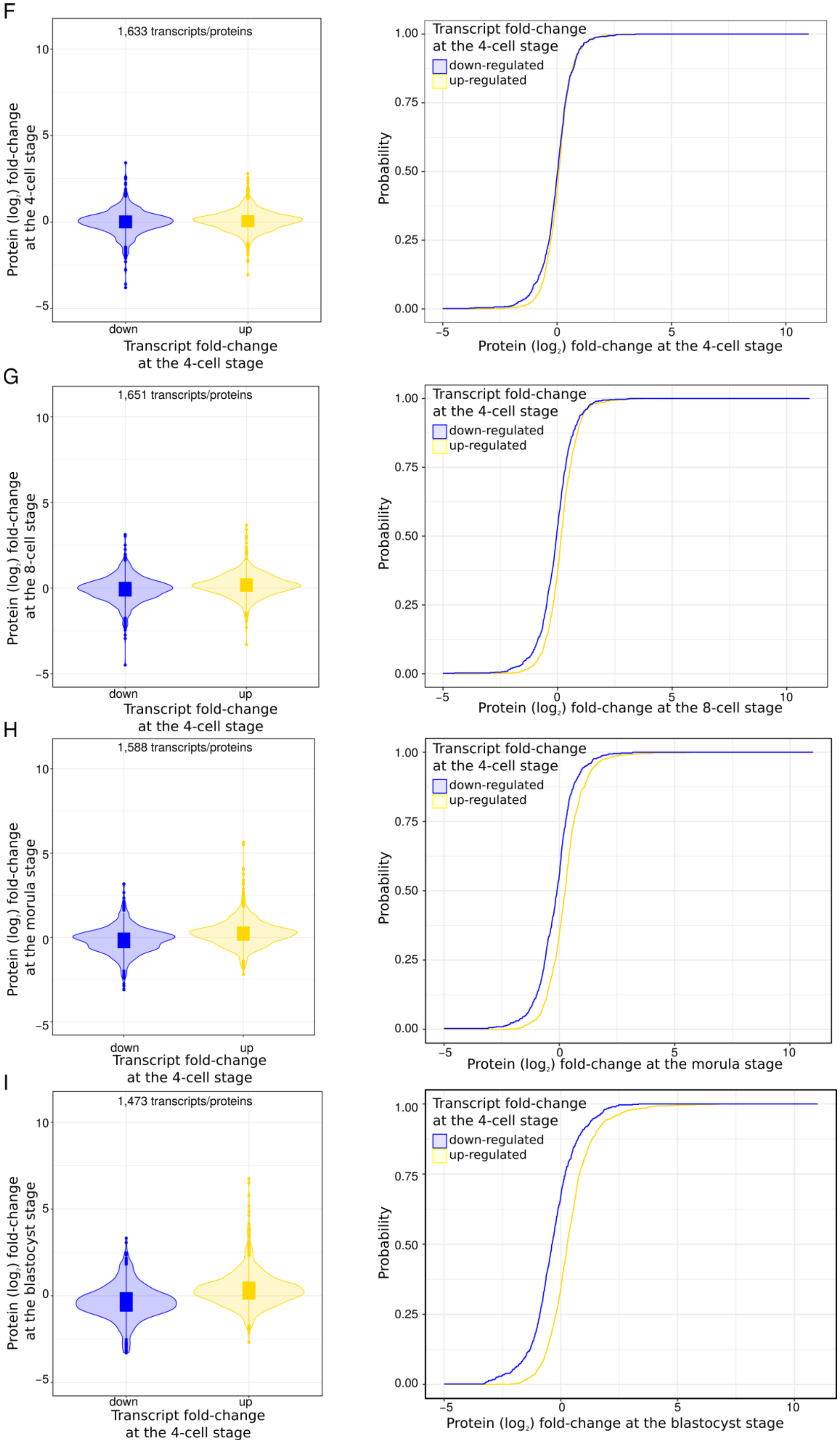
F
1,633 transcripts/proteins
Protein (log2) fold-change at the 4-cell stage
down
up
Transcript fold-change at the 4-cell stage
Transcript fold-change at the 4-cell stage
down-regulated
up-regulated
Probability
Protein (log2) fold-change at the 4-cell stage
G
1,651 transcripts/proteins
Protein (log2) fold-change at the 8-cell stage
down
up
Transcript fold-change at the 4-cell stage
Transcript fold-change at the 4-cell stage
down-regulated
up-regulated
Probability
Protein (log2) fold-change at the 8-cell stage
H
1,588 transcripts/proteins
Protein (log2) fold-change at the morula stage
down
up
Transcript fold-change at the 4-cell stage
Transcript fold-change at the 4-cell stage
down-regulated
up-regulated
Probability
Protein (log2) fold-change at the morula stage
I
1,473 transcripts/proteins
Protein (log2) fold-change at the blastocyst stage
down
up
Transcript fold-change at the 4-cell stage
Transcript fold-change at the 4-cell stage
down-regulated
up-regulated
Probability
Protein (log2) fold-change at the blastocyst stage

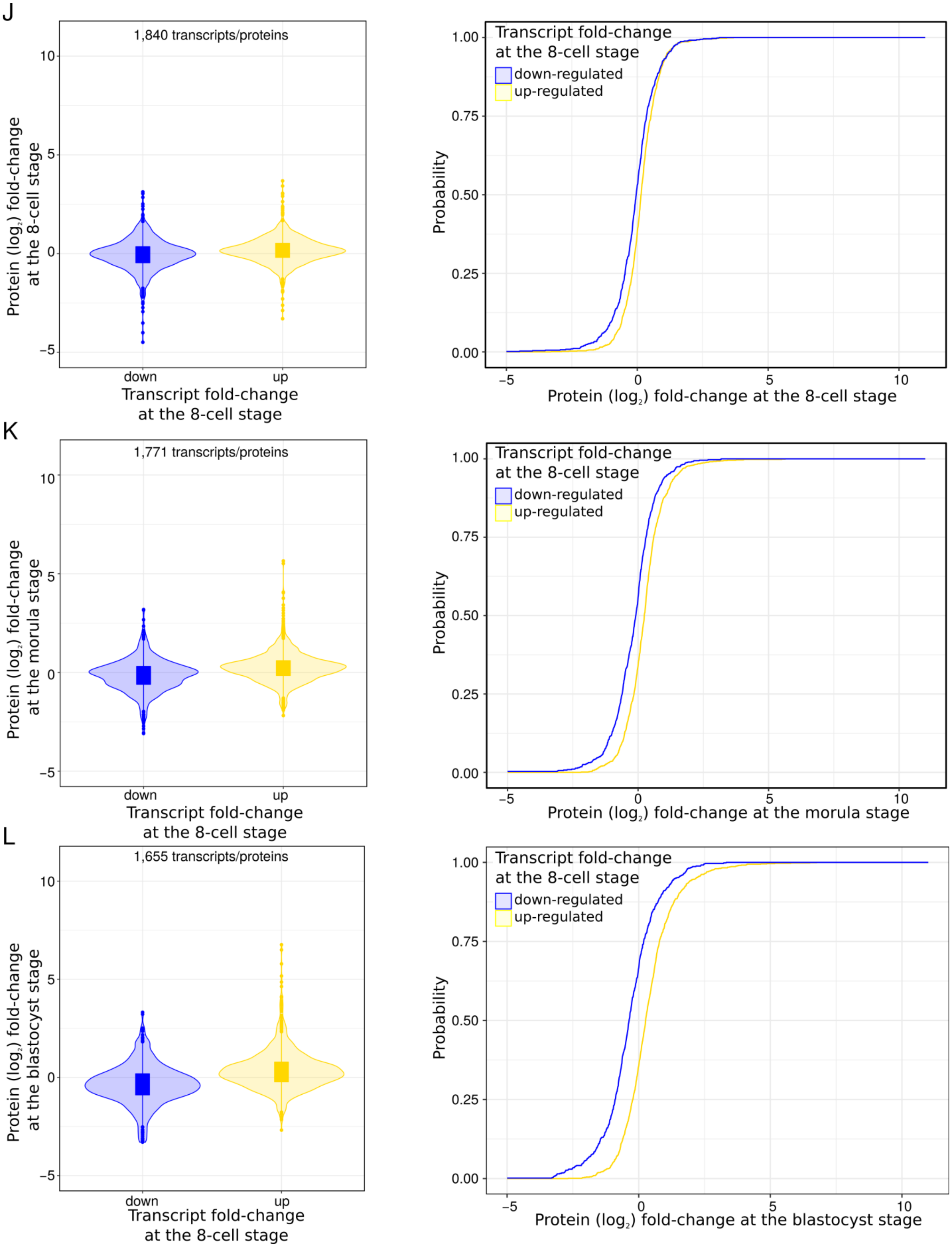
J
1,840 transcripts/proteins
Protein (log2) fold-change at the 8-cell stage
down
up
Transcript fold-change at the 8-cell stage
Transcript fold-change at the 8-cell stage
down-regulated
up-regulated
Probability
Protein (log2) fold-change at the 8-cell stage
K
1,771 transcripts/proteins
Protein (log2) fold-change at the morula stage
down
up
Transcript fold-change at the 8-cell stage
Transcript fold-change at the 8-cell stage
down-regulated
up-regulated
Probability
Protein (log2) fold-change at the morula stage
L
1,655 transcripts/proteins
Protein (log2) fold-change at the blastocyst stage
down
up
Transcript fold-change at the 8-cell stage
Transcript fold-change at the 8-cell stage
down-regulated
up-regulated
Probability
Protein (log2) fold-change at the blastocyst stage

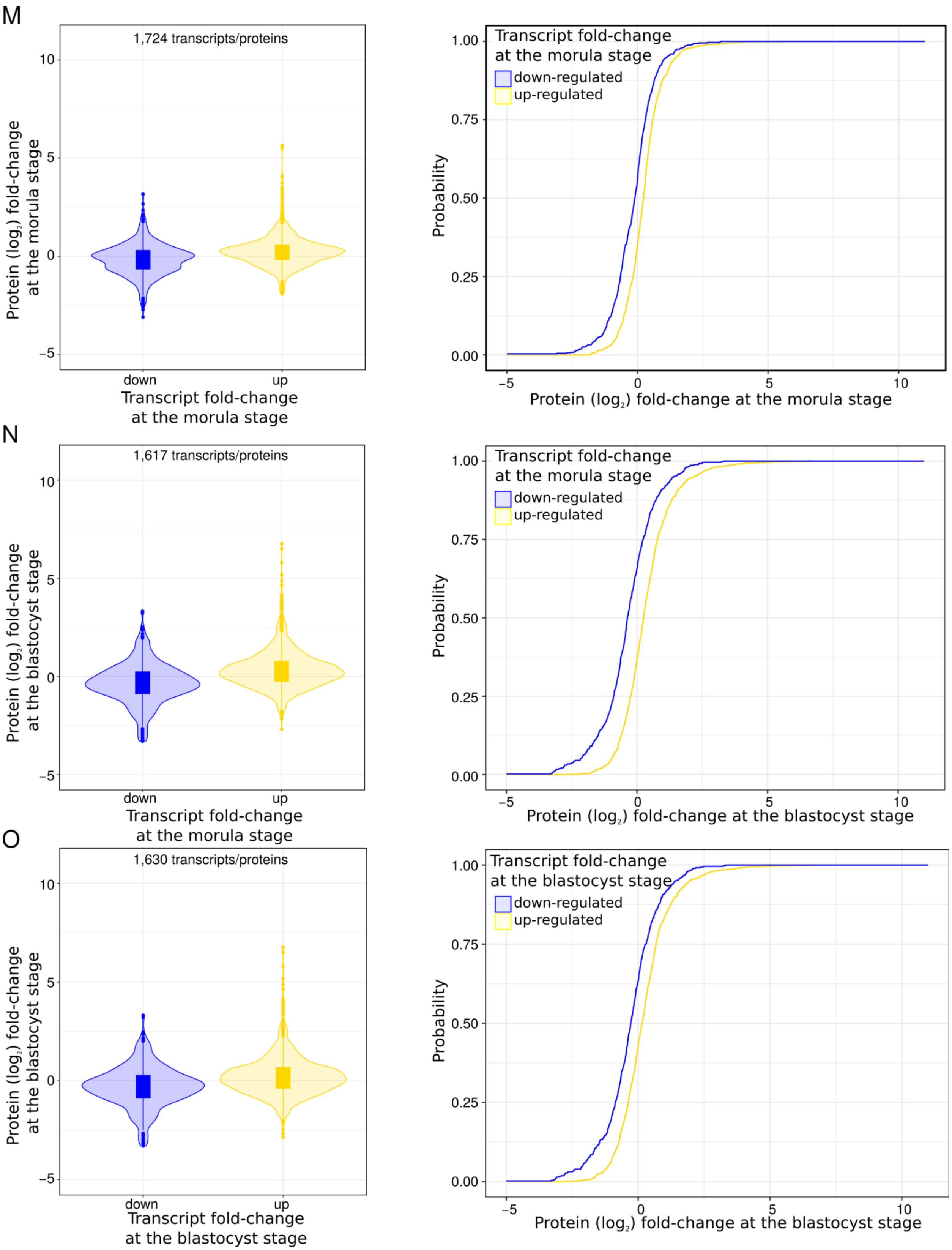

Supplemental Fig. S7. Changes in transcriptome are only globally reflected at the proteome level from the 16-cell stage onwards. Each panel (A-O) shows for a pair of developmental

stages (S_i, S_j) the distribution of protein ($\log_2$) fold-changes at stage S_j relative to the oocyte, for proteins whose cognate transcripts are down- (blue) or up- (yellow) regulated at stage S_i, relative to the oocyte. Left panels: Violin plots showing the distribution of protein ($\log_2$) fold-changes for proteins whose cognate transcripts are down- (blue) or up- (yellow). Right panels: Cumulative density functions (CDF) of the estimated density functions shown in left figure. Note, that in panels D, H, K and M as well as in panels E, I, L, N and O, which correspond to S_j morula and blastocyst, respectively, the CDF for the proteins whose transcripts are up-regulated is shifted to the right compared to the CDF for the proteins whose transcripts are down-regulated.

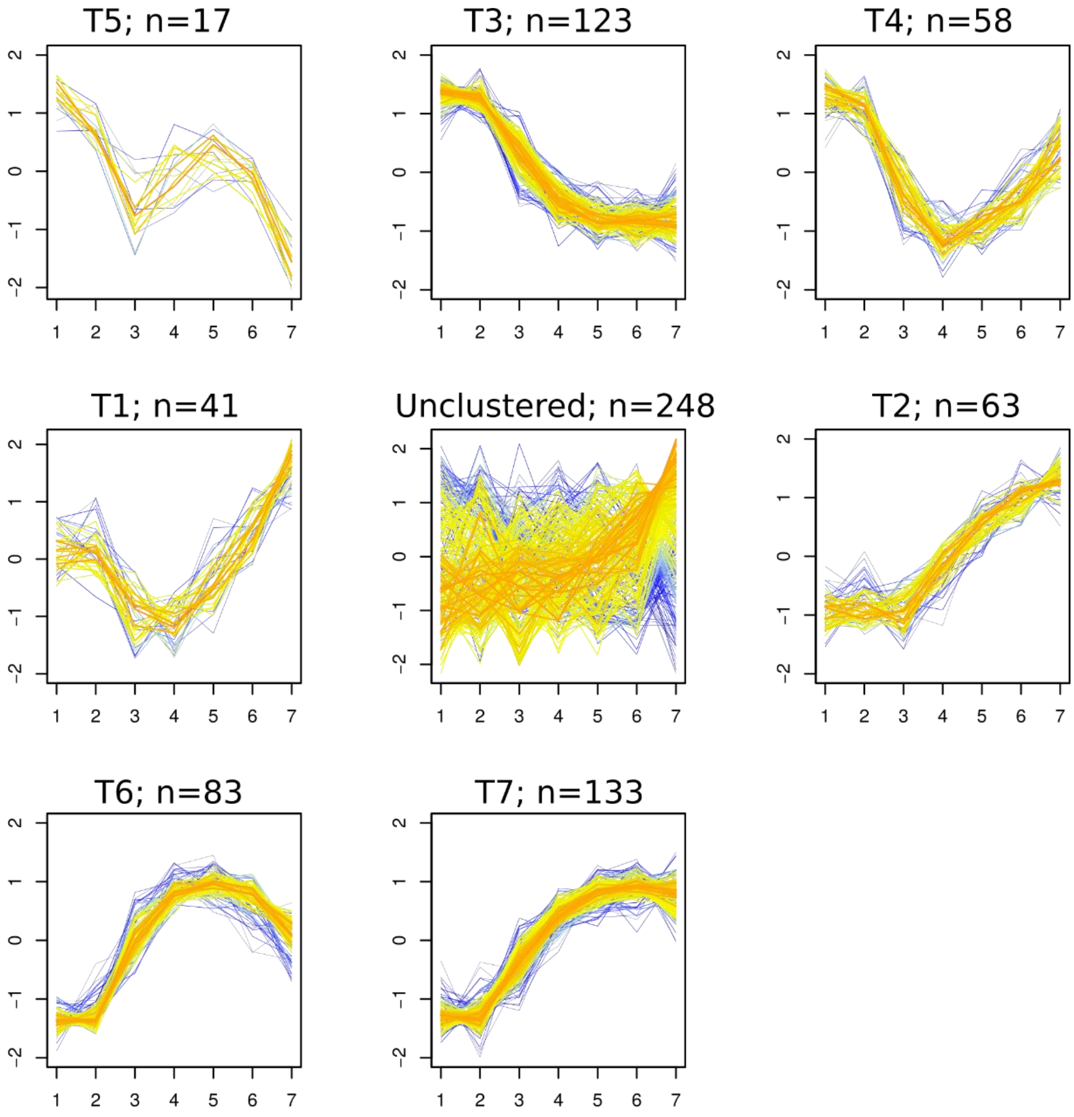

Supplemental Fig. S8. Transcript clusters. The x axis represents the seven developmental stages considered (1: oocyte, 2: 1-cell, 3: 2-cell, 4: 4-cell, 5: 8-cell, 6: morula and 7: blastocyst). The y axis represents the fold-change relative to the oocyte, expressed in terms of standard deviation. The coloring reflects the similarity of the temporal profile of a protein to the median profile of its cluster (red: more similar; blue: less similar).

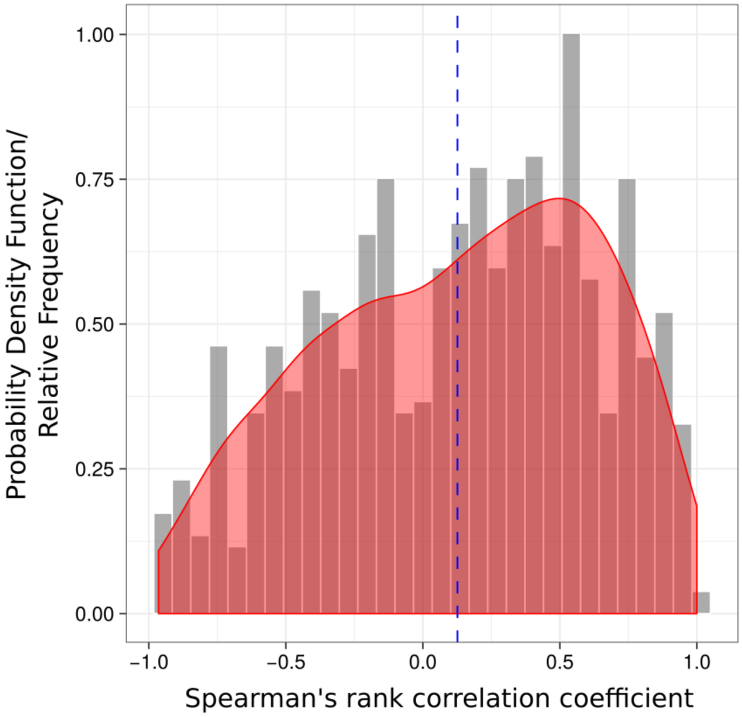

Supplemental Fig. S9. Scaled relative frequency histogram (gray bars) and estimated probability density function (red) for the Spearman's rank correlation coefficients computed between the transcript regularized log-transformed read counts and protein $\log_2$ L/H ratios across seven developmental stages for each gene. Seven hundred seventy-two genes corresponding to differentially expressed proteins were considered for this analysis. The blue dashed line shows the median.

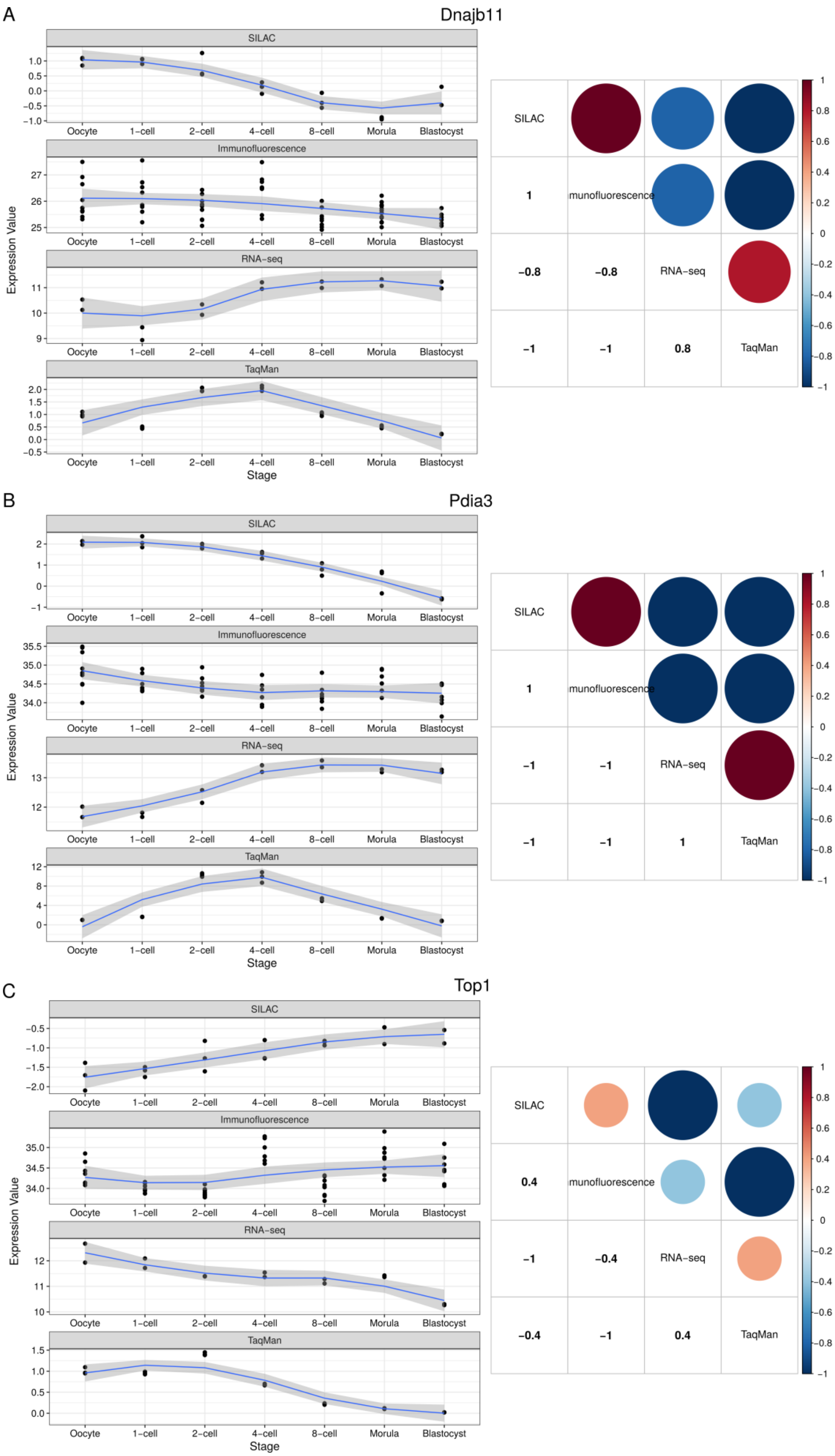
A
Dnajb11
SILAC
Immunofluorescence
RNA−seq
TaqMan
Expression Value
Oocyte 1−cell 2−cell 4−cell 8−cell Morula Blastocyst
Stage
SILAC
1 munofluorescence
−0.8 −0.8 RNA−seq
−1 −1 0.8 TaqMan
B
Pdia3
SILAC
Immunofluorescence
RNA−seq
TaqMan
Expression Value
Oocyte 1−cell 2−cell 4−cell 8−cell Morula Blastocyst
Stage
SILAC
1 munofluorescence
−1 −1 RNA−seq
−1 −1 1 TaqMan
C
Top1
SILAC
Immunofluorescence
RNA−seq
TaqMan
Expression Value
Oocyte 1−cell 2−cell 4−cell 8−cell Morula Blastocyst
Stage
SILAC
0.4 munofluorescence
−1 −0.4 RNA−seq
−0.4 −1 0.4 TaqMan

Supplemental Fig. S10. Validation of the SILAC and RNA-seq data using immunofluorescence and TaqMan assays, respectively. (left) Expression in the oocyte and six developmental stages. Data variation was smoothed using loess (with a span parameter of 1) curves (in blue) across the ranks corresponding to the developmental progression of the embryo starting from the oocyte, with 95% confidence interval (in grey). (right) Spearman's rank correlation coefficients (lower half) computed between the fitted loess protein/transcript profiles across the oocyte, 1-, 2- and 4-cell embryos as determined by SILAC, immunofluorescence, RNA-seq, and TaqMan assays. Transcriptomic measurements for early and late 2-cell stages were averaged. The correlation matrix was visualized using the R corrplot package [5]. The size and color of the circles (upper half) are both indicators of the magnitude of the correlation. A) Dnajb11; B) Pdia3; C) Top1.

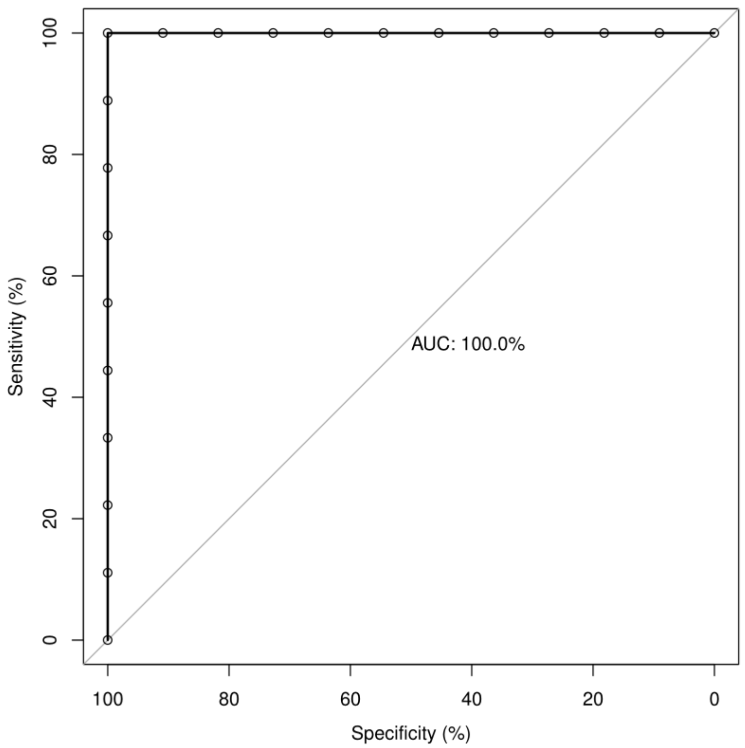

Supplemental Fig. S11. Prediction (binary classification) of early versus late developmental stages based solely on the protein expression values. Receiver Operating Characteristic (ROC) curves (area under the curve [AUC] value is indicated). The performance of the classifier was evaluated in a leave-one-out cross-validation (LOOCV) framework to reduce the risk of overfitting. The ROC was constructed using the posterior probabilities of the LDA for each of the test samples. Each point on the ROC curve represents a sensitivity/specificity pair corresponding to the posterior probability of a particular test sample. The AUC of 1.00 indicates that the LDA classifier is perfectly able to separate early from late developmental stages.

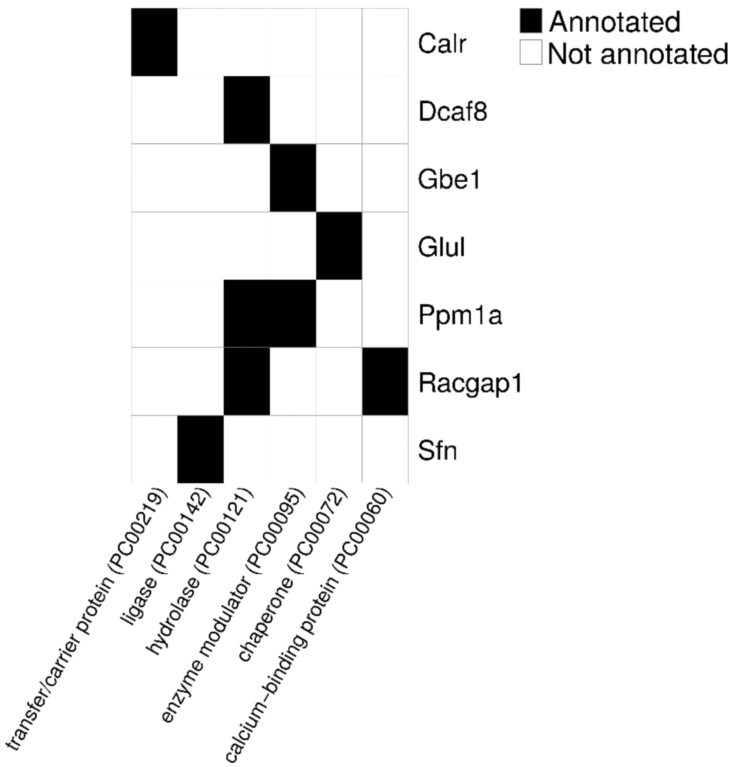

Supplemental Fig. S12. PANTHER14.1 protein classification available for seven of the twenty candidate proteins markers.

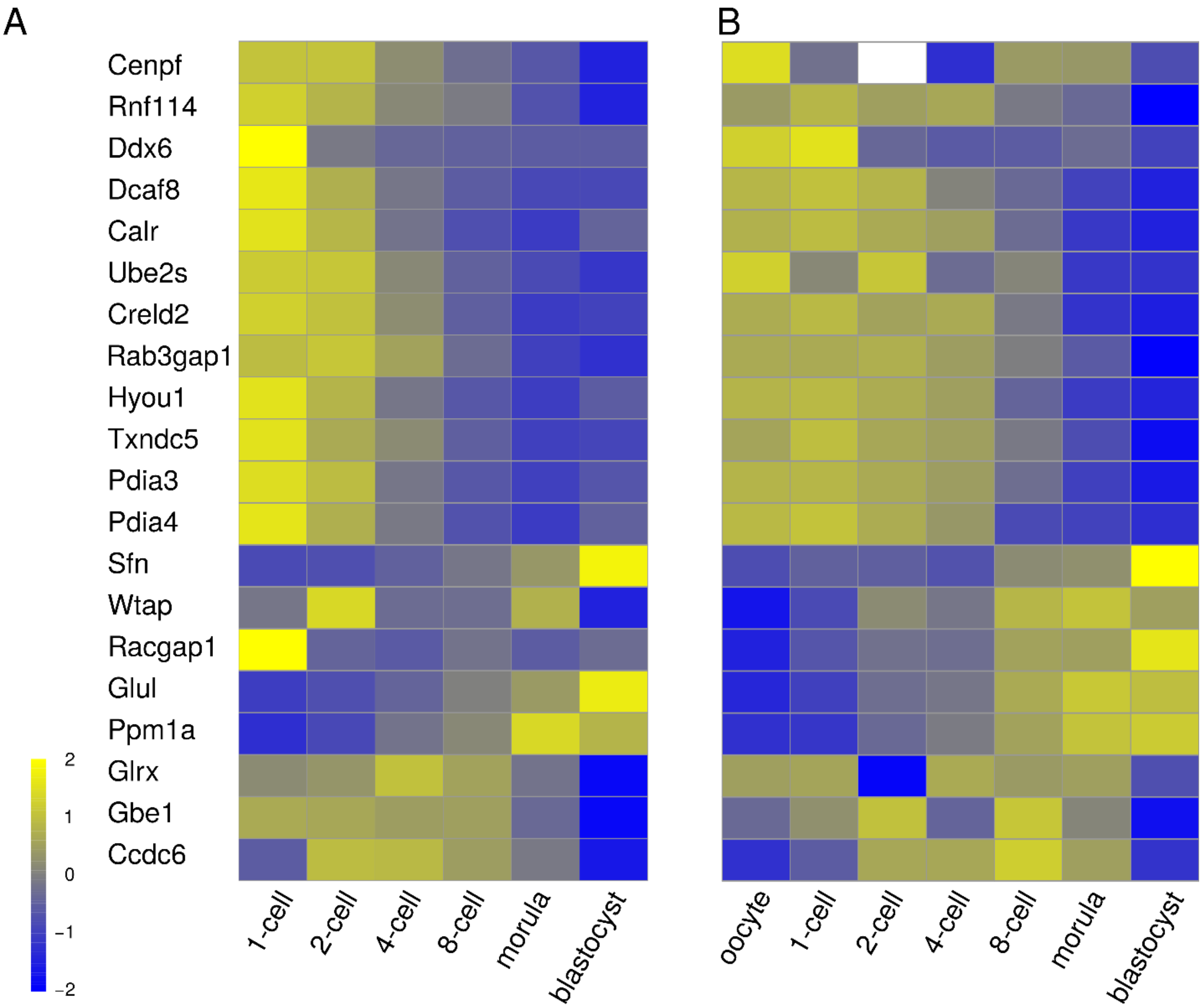

Supplemental Fig. S13. Validation of twenty candidate developmental protein markers. Heatmaps representing the expression of the markers across multiple developmental stages. Rows have been scaled to have a mean of zero and standard deviation equal to 1. The scale and names of the markers are only indicated for panel A), but shared across all panels. A) Normalized protein ratios from the dataset published by Gao et al. (2017). Note that these authors did not quantify protein expression in the oocyte. B) Log_2-transformed LH protein ratios from own SILAC dataset generated in absence of hormone stimulation.

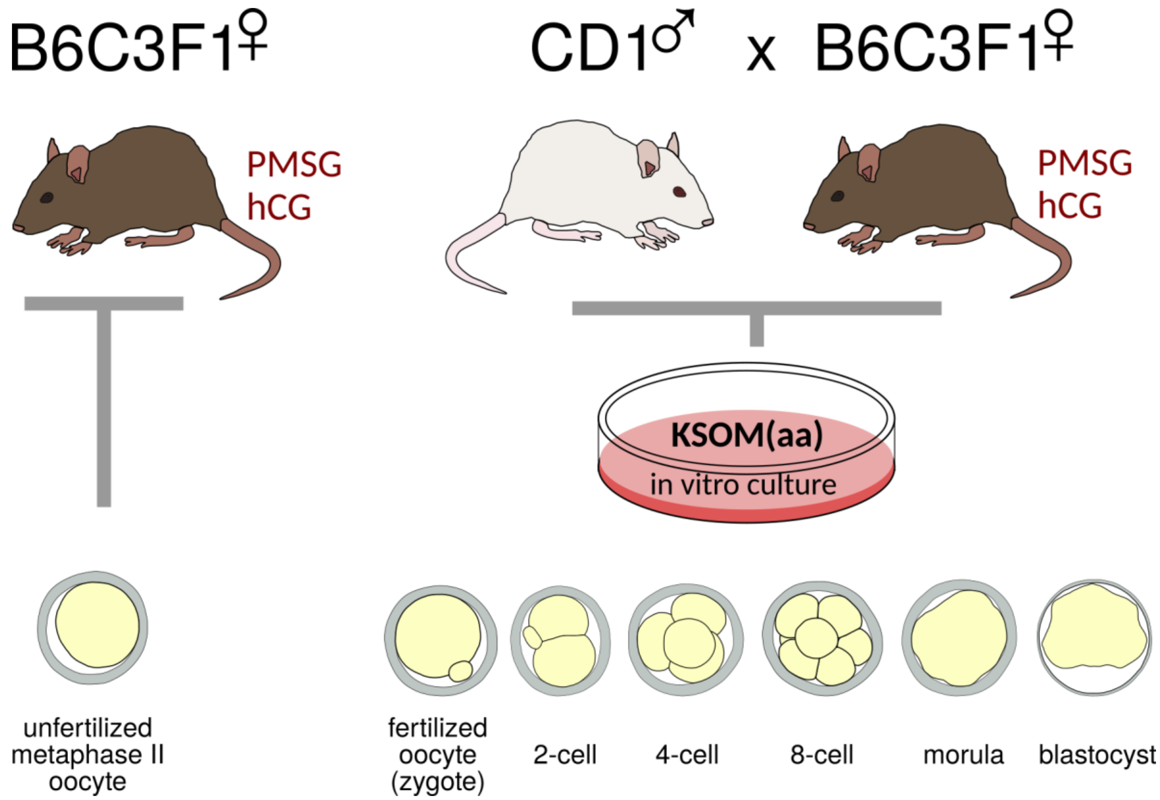

Supplemental Fig. S14. *In vivo*-fertilized, *in vitro*-cultured mouse oocytes as a source of embryonic material for proteomic analysis. Metaphase II or pronuclear-stage oocytes were collected from the oviducts of superovulated females, and the latter were cultured in KSOM (Potassium simplex optimized medium) medium with aminoacids to collect embryonic stages at specific time points, up to blastocyst. PMSG, pregnant mare's serum gonadotropin. hCG, human chorionic gonadotropin.

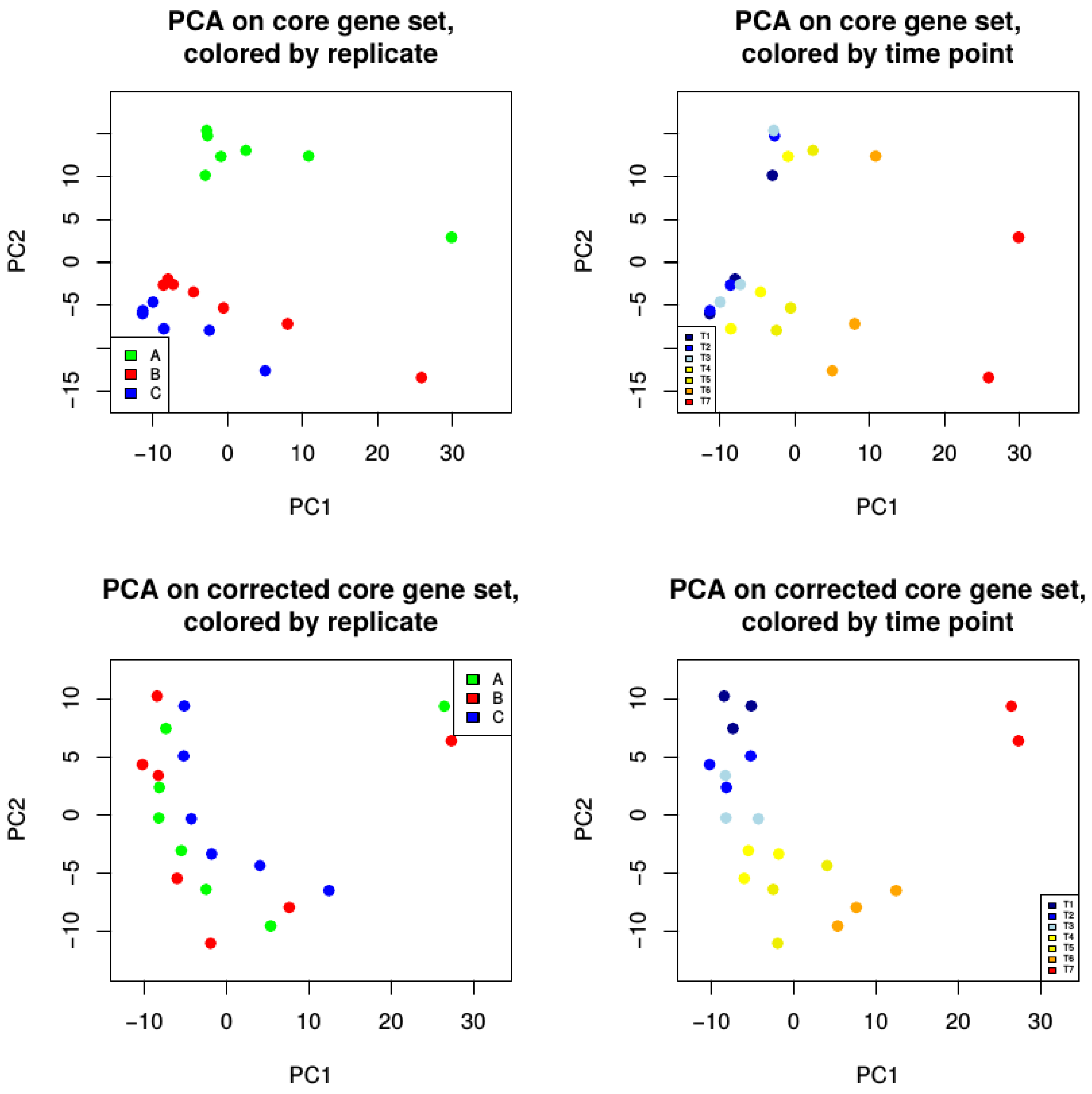

Supplemental Fig. S15. Protein dataset batch correction. Principal Component Analysis (PCA) on the proteome dataset, based on the 1,709 proteins detected in all replicates of all developmental stages. The upper panels visualize the first two PCs before applying batch correction, the lower panels visualize them after applying batch correction. In the left panels, samples are colored according to the replicate they belong to, in the right panel they are colored according to stage (T1: oocyte; T2: 1-cell; T3: 2-cell; T4: 4-cell; T5: 8-cell; T6: morula; T7: blastocyst). In the upper panels, the high contribution of the replicate identity on total variation is clearly visible, motivating the application of a batch correction procedure, resulting in a

corrected visualization of the PCs that reflects the temporal progression from the oocyte to the blastocyst stage.

Supplementary Table Legends

Supplemental Table S1. Functional analysis of proteins that are differentially expressed between pairs of consecutive developmental stages ($\log_2$ fold-change ≥1 or ≤−1 between any two developmental stages, P-value≤0.05 from ANOVA). The detected proteome was used as background for the analysis. Significantly enriched (FDR-adjusted P-value<5%) gene ontology terms (GOTERM), pathways (KEGG_PATHWAY) and keywords (KEYWORDS) were identified with DAVID (http://david.abcc.ncifcrf.gov/). The columns in the table contain information from the DAVID Functional Annotation Chart Report: Category (source provenance for the Term); Term (gene set name); Count (number of genes associated with this gene set); P-value (modified Fisher Exact P-value); List Total (number of genes in your query list mapped to any gene set in this ontology); Pop Hits (number of genes annotated to this gene set on the background list); Pop Total (number of genes on the background list mapped to any gene set in this ontology); and FDR.

Supplemental Table S2. Functional analysis of the protein clusters. The detected proteome (see Supplemental Table S5) was used as background for the analysis.

Supplemental Table S3. Immunofluorescence and enzymatic validation of protein abundances.

Supplemental Table S4. Functional analysis of the transcript clusters. The detected transcriptome was used as background for the analysis.

Supplemental Table S5. Validation of twenty candidate developmental protein markers. Computed across the expression profiles of the markers across multiple developmental stages with two independent datasets: Gao et al. (2017) and own SILAC dataset generated in absence of hormone stimulation.

Supplemental Table S5. Batch-corrected quantile-normalized protein L/H ratios for all samples in this study.

References

1 Gao, Y. *et al.* Protein Expression Landscape of Mouse Embryos during Pre-implantation Development. *Cell Rep* **21**, 3957-3969, doi:10.1016/j.celrep.2017.11.111 (2017).

2 Mi, H. *et al.* PANTHER version 11: expanded annotation data from Gene Ontology and Reactome pathways, and data analysis tool enhancements. *Nucleic Acids Res* **45**, D183-D189, doi:10.1093/nar/gkw1138 (2017).

3 Mi, H., Muruganujan, A., Casagrande, J. T. & Thomas, P. D. Large-scale gene function analysis with the PANTHER classification system. *Nat Protoc* **8**, 1551-1566, doi:10.1038/nprot.2013.092 (2013).

4 Lex, A., Gehlenborg, N., Strobelt, H., Vuillemot, R. & Pfister, H. UpSet: Visualization of Intersecting Sets. *IEEE Trans Vis Comput Graph* **20**, 1983-1992, doi:10.1109/TVCG.2014.2346248 (2014).

5 R package "corrplot": Visualization of a Correlation Matrix (Version 0.84) (2017).
